# Computational and Mass Spectrometry-Based Approach Identify Deleterious Non-Synonymous Single Nucleotide Polymorphisms (nsSNPs) in JMJD6

**DOI:** 10.3390/molecules26154653

**Published:** 2021-07-31

**Authors:** Tianqi Gong, Lujie Yang, Fenglin Shen, Hao Chen, Ziyue Pan, Quanqing Zhang, Yan Jiang, Fan Zhong, Pengyuan Yang, Yang Zhang

**Affiliations:** 1 Department of Systems Biology for Medicine, Institutes of Biomedical Sciences, Shanghai Medical College, Fudan University, Shanghai 200032, China; 16111510013@fudan.edu.cn (T.G.); 18111510035@fudan.edu.cn (L.Y.); shenfenglin@fudan.edu.cn (F.S.); 19211360002@fudan.edu.cn (Z.P.); jiangyan515@fudan.edu.cn (Y.J.); 2 College of Bioscience and Biotechnology, Yangzhou University, Yangzhou 225009, China; chen33hao@163.com; 3 Department of Chemistry, University of California, Riverside, CA 92521, USA; quanqinz@ucr.edu

**Keywords:** JMJD6, deleterious nsSNPs, computational workflow, systematic filtration

## Abstract

The jumonji domain-containing protein 6 (JMJD6) gene catalyzes the arginine demethylation and lysine hydroxylation of histone and a growing list of its known substrate molecules, including p53 and U2AF65, suggesting a possible role in mRNA splicing and transcription in cancer progression. Mass spectrometry-based technology offers the opportunity to detect SNP variants accurately and effectively. In our study, we conducted a combined computational and filtration workflow to predict the nonsynonymous single nucleotide polymorphisms (nsSNPs) present in JMJD6, followed by a liquid chromatography-tandem mass spectrometry (LC-MS/MS) analysis and validation. The computational approaches SIFT, PolyPhen-2, SNAP, I-Mutant 2.0, PhD-SNP, PANTHER, and SNPS&GO were integrated to screen out the predicted damaging/deleterious nsSNPs. Through the three-dimensional structure of JMJD6, H187R (rs1159480887) was selected as a candidate for validation. The validation experiments showed that the mutation of this nsSNP in JMJD6 obviously affected mRNA splicing or the transcription of downstream genes through the reduced lysyl-hydroxylase activity of its substrates, U2AF65 and p53, further indicating the accuracy of this prediction method. This research provides an effective computational workflow for researchers with an opportunity to select prominent deleterious nsSNPs and, thus, remains promising for examining the dysfunction of proteins.

## 1. Introduction

Chromosome 17q21-ter gains were well-characterized as common gene copy number variations, and its chromosomal inversions were implicated as important genetic factors of tumor progression in human lung, liver, neuroblastoma, breast cancer, and stomach cancer [1,2,3,4,5,6,7]. The jumonji domain-containing protein 6 (JMJD6) gene, located at chromosome 17qter, has a dual function of lysyl hydroxylase and arginine demethylase. As a lysyl hydroxylase, JMJD6 catalyzes U2AF65 by a posttranslational lysyl-5-hydroxylation [6,8], as well as being physically associated with p53, and catalyzes the p53 protein hydroxylation via modifying its C-terminal domain, resulting in negatively regulated p53 activity. It has been well-studied that p53 conducts an important role in many processes, such as regulation of the cell cycle, differentiation, apoptosis, metabolism, and cell reprogramming, and U2AF65 functions as a positive regulator in alternative splicing cell proliferation [9,10,11]. On the other hand, JMJD6 forms a protein complex with BRD4 and the demethylates histone H4 at arginine 3 (H4R3) of the anti-pause enhancer of the target gene, thereby upregulating the target gene transcriptionally, resulting in the release of RNA polymerase II (RNA Pol II) from the pause near the promoter. These findings revealed the comprehensive role of JMJD6 in cell biology. 

Nonsynonymous single nucleotide polymorphisms (nsSNPs), single-base changes to the amino acid sequence of its encoded protein, have been well-studied since, nsSNP variations are associated with disease via abolishing the original functionality of individual proteins—for example, stability and enzyme active sites. With the rapid advancement of DNA sequencing and genotyping technology, millions of SNPs have been discovered. Most nsSNPs are neutral or harmless, with little influence on the protein function, while some deleterious nsSNPs are known to be associated with genetic or complex diseases [12,13]. International initiatives, such as the 1000 Genomes Project [14] and the Exome Sequencing Project (http://evs.gs.washington.edu/EVS/, accessed on 17 June 2020), as well as large-scale studies such as GWAS (GWAS Genome-Wide Association Studies) [15] and WES (Whole-genome/Exome Sequencing approaches) [16], promote investigations of disease-causing and phenotype-related genetic variations through uncovering the genetic variations between individuals. 

Currently, many computational tools have been developed for predicting deleterious nsSNPs based on different algorithms, with large numbers of disease-related variants being discovered [17,18,19]. These computational algorithms mainly involve predicting potential structural and functional impacts caused by variants. However, it is not sufficient to rely solely on calculations for predictions. The relationship between the molecular structure and site function cannot be ignored. Through integrating computational approaches and biological filtration, it is very effective and accurate to screen out nsSNP candidates for further biological discussions and downstream mechanism exploration. 

Recently, technological advancements in mass spectrometry-based high-throughput proteomics have redefined the biomedical sciences and have played a key role in understanding human diseases. State-of-the-art MS-based shotgun proteomics has been developed rapidly and are sufficiently powerful to interrogate protein variations with high resolution and sensitivity [20,21]. For example, the rapid detection of mutations in protein-coding genes and the impact of these mutations on the protein structure, function, and interacting protein ligands have been studied [22,23,24,25,26,27].

Taking these into consideration, the main objective of the present study was to predict the possible deleterious nsSNPs of JMJD6 to investigate their effects on the downstream functions of the JMJD6 protein, as well as to provide a combined computational workflow for more researchers. Consequently, we integrated multiple prediction tools to investigate potential deleterious nsSNPs in JMJD6. The nsSNP H187R (rs1159480887), presented in the JmjC domain of JMJD6 (174-288 amino acid residues), shows 100% sequence conservation in multiple species and was found to be more meaningful in a three-dimensional structure. To our knowledge, this is the first comprehensive in silico study for the analysis of JMJD6 nsSNPs effects on the protein function and structure.

## 2. Results

### 2.1. Deleterious nsSNPs Predicted in JMJD6

**Figure 1:**
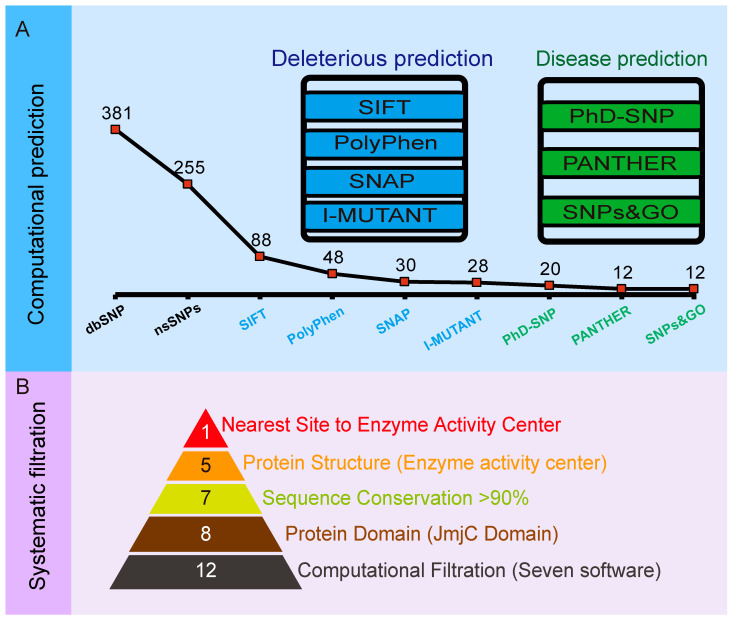
Schema of screening deleterious nsSNPs in JMJD6. (**A**) Computational workflow to predict deleterious nsSNPs in JMJD6. A total of 381 SNPs were retrieved from dbSNP for protein JMJD6. Firstly, SIFT, PolyPhen-2, and SNAP were conducted to predict deleterious nsSNPs based on the sequence homology and structural homology and I-Mutant 2.0 to predict the protein stability changes upon single-site mutations. Secondly, disease-associated SNPs were inferred using PhD-SNP, SNPs&GO, and PANTHER. After that, 12 candidate deleterious nsSNPs were derived by the union of seven public free nsSNP prediction softwares. (**B**) A schema of the comprehensive and systematic screening of nsSNPs in JMJD6. Under consideration of the JmjC domain and sequence conservation, as well as the three-dimensional structure of JMJD6, 5 nsSNPs (H187R, K204E, F266S, P268T, and P268A) were filtered out, and H187R was located nearest to the enzyme activity center.

A total of 381 SNPs were retrieved from dbSNP for protein JMJD6; out of which, 255 (66.9%) were nsSNPs (Table S1). To determine the structural and functional effects and to screen out the most promising deleterious nsSNPs of the 255 SNPs in the JMJD6 protein structure, we conducted a three-step multilayer approach (Figure 1). Step 1 aimed at predicting deleterious nsSNPs based on sequence homology and structural homology mainly by SIFT, PolyPhen-2, and SNAP and to predict the protein stability changes upon single-site mutations by I-Mutant 2.0 (Figure 1A). Step 2 was performed to identify and infer disease-associated SNPs using PhD-SNP, SNPs&GO, and PANTHER (Figure 1A). In step 3, we further nominated crucial nsSNPs via structural feature-based functional filtration (Figure 1B) and then conducted biochemical experiments to explore how the mutation of JMJD6 (H187R) regulates the modification of its substrates.

In step 1 (Figure 1A), we first conducted a SIFT prediction based on the alignment of the homologous protein sequences and predicted the effect of amino acid substitutions on the protein function. The retrieved nsSNPs from the dbSNP database were submitted to SIFT, and 88 deleterious nsSNPs were shown to be “AFFECT PROTEIN FUNCTION”, with probability scores ≤0.05. Then, the 88 selected deleterious nsSNPs were then further analyzed based on the protein structure and function using PolyPhen-2, a sequence-based approach. The predictions of the damaging nsSNPs were categorized on three different levels in order from high to low confidence: probably damaging, possibly damaging, and benign (meaning tolerant). Among them, 28 nsSNPs were predicted as “benign”, 12 nsSNPs were predicted as “possibly damaging”, and 48 nsSNPs were predicted as “probably damaging” (deleterious). It is well-known that most disease-related nsSNPs affect the protein stability. Thus, we conducted the SNAP approach to predict the effect of a single amino acid substitution on the mutant protein. The 48 deleterious nsSNPs were further verified by SNAP to classify nsSNPs as non-neutral (effect on function) and neutral (no effect) using sequence-based computationally acquired information alone, and 30 nsSNPs were identified as “deleterious” (Table 1). After that, I-Mutant 2.0 was used to study the effects of these variants on the stability of the protein. It determined the change in free energy and gave the direction for the change. All the nsSNPs except rs370497426 (Y63C) and rs1403684218 (K204M) were found to have a decrease in their stability due to these single-point mutations, resulting in a list of 28 deleterious nsSNPs (Table 1). 

### 2.2. Inferences of Damaging Effect of Disease-Related nsSNPs Using Multiple Approaches

Subsequently, the 28 predicted nsSNPs listed in Table 1 were uploaded to PhD-SNP, PANTHER, and SNP&GO, respectively (Figure 1B). 

PhD-SNP and SNP&GO were used to analyze the mutants. They are both support vector machine (SVM)-based classifiers to evaluate the probability of the amino acid being deleterious. They derived features like the protein sequence, 3D structure, protein sequence profile, and protein function. Twenty nsSNPs were determined as “deleterious” from PhD-SNP, and 12 nsSNPs were determined as “deleterious” in SNP&GO (Table 1). The specific purpose of using two approaches to discriminate disease-related nsSNPs is to resolve such biases in the nsSNP classification and to increase the confidence of the predictive analysis, since they perform similar algorithms.

PANTHER, a model that uses the evolutionary conservation of amino acids to predict pathogenic coding variants, identified 20 nsSNPs as “deleterious”, 6 as “Neutral”, and the rest were “Not aligned”. Based on the results above, 12 top disease-associated nsSNPs (F99C, P129L, F156S, D159N, R181C, H187R, K204E, R242W, F266S, P268T, P268A, and I284T) were selected for further study (Table 1). The whole process of filtering deleterious nsSNPs in JMJD6 is shown in Figure 1.

### 2.3. Structural Feature-Based Functional Analysis Nominated Crucial nsSNPs

**Figure 2:**
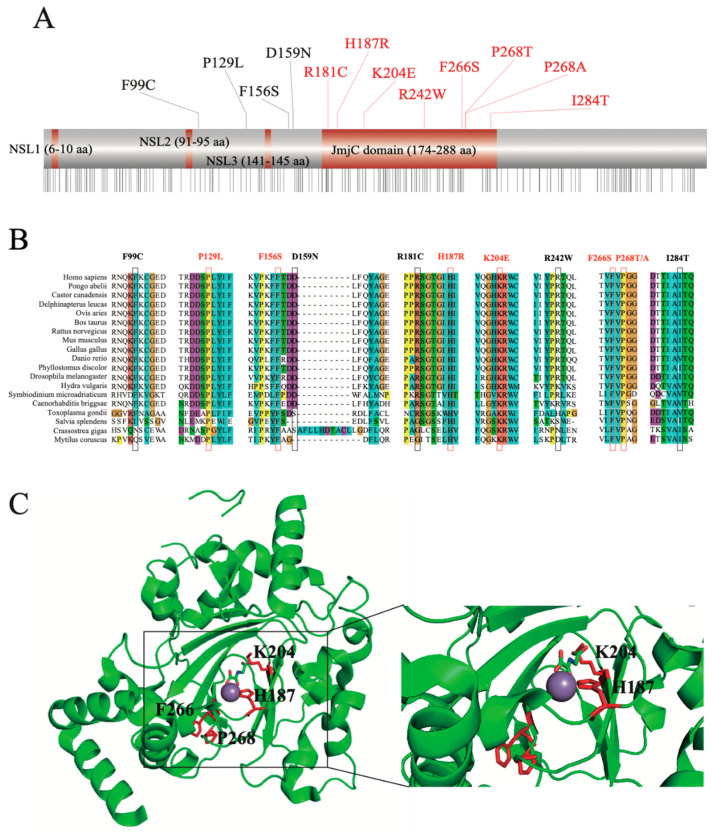
Protein homology and structural features. (**A**) Schematic representation of the JMJD6 protein. Eight mutations located at the JmjC domain (174-288 aa). (**B**) Multiple sequence alignment of the JMJD6 protein. Different colors represent different amino acids. Highly conserved mutation sites (consensus score > 90) are labeled in red (P129, F156, H187, K204, F266, and P268). (**C**) Crystal structure of JMJD6. H187 and K204 are located in the JMJD6 enzyme activity region, and H187 is the nearest site to the enzyme activity center. The purple ball represents Fe(II).

To explore the function-associated nsSNPs, we aligned the mutation sites to the JMJD6 protein sequence from the UniProt database (Figure 2A). Sequence-based structural-functional filtration showed that 8/12 nsSNPs, including R181C, H187R, K204E, R242W, F266S, P286T/A, and I284T, were present in the JmjC domain of the JMJD6 protein (174-288 amino acid residues). Then, we compared the degree of sequence conservation across the aligned sequences (Figure S1) using a panel of JMJD6 orthologs with various divergent levels from highly homologous sequences such as mammal orthologs, including humans (*Homo sapiens*), mice (*Mus musculus*), cattle (*Bos taurus*), Beluga whales (*Delphinapterus leucas*), rats (*Rattus norvegicus*), pale spear-nosed bats (*Phyllostomus discolor*), and Sumatran orangutans (*Pongo abelii*), to highly divergent ortholog sequences, including a JMJD6 ortholog from *Caenorhabditis briggsae*, *Symbiodinium microadriaticum*, and *Hydra vulgaris*. As shown in Figure 2B, the nsSNPs P129L, F156S, H187R, K204E, F266S, and P268T/A were highly conserved in all the organisms.

Since the crystal structure of JMJD6 is now available (PDB entry 6FQC), we decided to observe the six highly conserved nsSNP locations (Figure 2C). It was observed that both H187R and K204E are located in the JMJD6 enzyme activity region and play an important role in binding 2-OG (Figure 2C). Furthermore, H187R is located at the nearest enzyme activity center. These results confirmed previous predictions [28]. It was demonstrated that JMJD6 acts as an ɑ-ketoglutarate- and Fe (II)-dependent lysyl-hydroxylase to catalyze the substrate hydroxylation [29]. We hypothesized that JMJD6 (H187R) loses its lysyl-hydroxylase activity and attenuates the hydroxylation of substrates such as U2AF65 [8] and p53 [29].

Protein structure homology-modeling of the JMJD6 mutation (H187R) compared with that of the WT (Figure S2) demonstrated changes from a smaller His to a larger Arg at the 187 position without changing the charge (positive charge). Based on the crystal structure, we inferred that the H187R substitution acts to tweak the substrate conformation, and the residue has the potential to affect the “substrate pocket” of the active center of the enzyme.

### 2.4. JMJD6 (H187R) Abolishes Lysyl-Hydroxylation to U2AF65 and Influences mRNA Splicing

**Figure 3:**
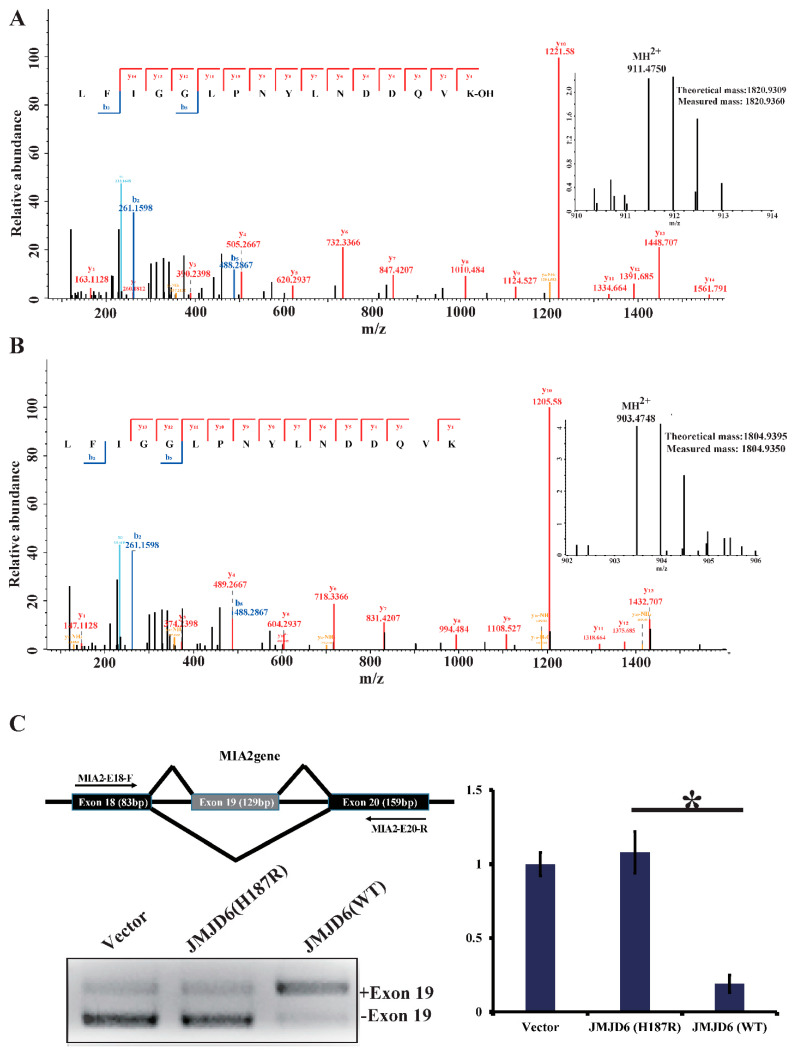
JMJD6 (H187R) abolishes the lysyl-hydroxylation of U2AF65 and influences mRNA splicing. (**A**,**B**) MS/MS analysis of recombinant U2AF65 reveals the hydroxylation of Lys-276 (**A**) by JMJD6 (WT) and no hydroxylation of Lys-276 (**B**) by JMJD6 (H187R). Recombinant U2AF65 was incubated with recombinant JMJD6 or recombinant JMJD6 (H187R) in the presence of 2-OG (500 µM) and Fe(II) (400 µM) for 2 h at 37 °C. The assay mixture was separated in SDS-PAGE, and the band corresponding to the molecular weight of GST-U2AF65 was excised, digested, and then analyzed. Data for the hydroxylated peptides (top) and unmodified ones (bottom) are shown. Inserts show the MH2+ peptide precursor ions that were fragmented. (**C**) Jmjd6 regulates the alternative splicing. Exon structure of the MIA2 gene showing exons (18–20) in boxes, introns as horizontal lines, and splicing patterns as diagonal lines. RT-PCR analyses detecting endogenous MIA2 RNA from HeLa cells transfected with pcDNA3.1, JMJD6 (WT), or JMJD6 (H187R). Histogram on the right shows the mean amount of –ex19-spliced isoform analyzed through quantitative PCR from three independent experiments (* *p* < 0.05).

To investigate our hypothesis that JMJD6 (H187R) impairs its lysyl-hydroxylase activity to U2AF65 (Figure S3), we first incubated bacterially purified GST-U2AF65 with GST-JMJD6 (WT) or GST-JMJD6 (H187R) in the presence of Fe(II) and α-ketoglutarate (2-OG) for 2 h at 37 °C. The reaction mixture was then resolved on SDS-PAGE and stained with Coomassie brilliant blue (CBB). The protein bands on the gels were retrieved and analyzed by liquid chromatography-tandem mass spectrometry (LC-MS/MS). The LC-MS/MS analysis revealed a +16-Dalton mass shift for lysine (K) 276 of the U2AF65 protein incubated with JMJD6 (WT) from HeLa cells (Figure 3A); no hydroxylation of U2AF65 was detected when incubated with JMJD6 (H187R) (Figure 3B). The detailed peptide lists are in Table S2. These results support an argument that the 187aa mutation of JMJD6 abolishes its 2-OG- and Fe (II)-dependent lysyl-hydroxylase to catalyze U2AF65 hydroxylation. 

U2AF65 is a protein important for pre-mRNA splicing [30]. The modulation of splice site recognition by U2AF65 was proven to influence alternative splicing [8,31]. To investigate whether this mutant influences the U2AF65 function because of the abolishment of hydroxylation, we detected an alternative splicing pattern of the endogenous tumor antigen gene MIA2, which is regulated by the splicing regulatory protein SRSF1. The RT-PCR analyses detected endogenous MIA2 RNA from HeLa cells transfected with pcDNA3.1, JMJD6 (WT), or JMJD6 (H187R). It is shown that, compared to JMJD6 (WT), JMJD6 (H187R) altered the alternative splicing pattern of the MIA2 gene (Figure 3C), showing an elevated amount of exon 19 skipping (–19) of the MIA2 gene (* *p* < 0.05).

### 2.5. JMJD6 (H187R) Abolishes Lysyl-Hydroxylation to p53 and Can’t Inhibit p21 Expression

**Figure 4:**
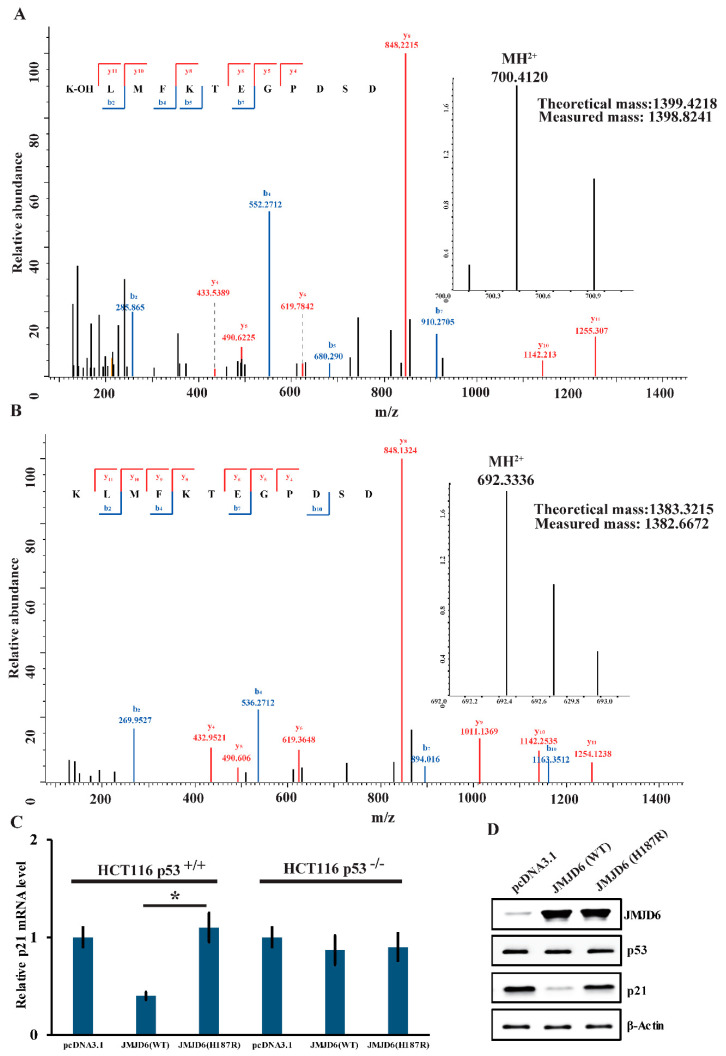
JMJD6 (H187R) abolishes the lysyl-hydroxylation of p53 and cannot inhibit p21 expression. (**A**,**B**) MS/MS analysis of recombinant p53 reveals the hydroxylation of Lys-382 (**A**) by JMJD6 (WT) and no hydroxylation of Lys-382 (**B**) by JMJD6 (H187R). Recombinant p53 was incubated with recombinant JMJD6 (WT) or recombinant JMJD6 (H187R) in the presence of 2-OG and Fe(II). The mixture was then separated on SDS-PAGE, and the band corresponding to the molecular weight of p53 was excised and digested with trypsin and analyzed by LC-MS/MS. (**C**,**D**) The regulation of p53 transcriptional activity by JMJD6 (WT) or JMJD6 (H187R). Measurement of the mRNA (**C**) and protein (**D**) levels of p21 by real-time RT-PCR and Western blotting in the HCT116 cells that were transfected with JMJD6 (WT) or JMJD6 (H187R). Each bar represents the mean ± standard variations for triplicate measurements. * *p* < 0.05.

To gain further support of the results above, we also detected the lysyl-hydroxylation of p53 by JMJD6 (WT) or JMJD6 (H187R). We obtained that there was a +16-Dalton mass shift for lysine (K) 382 of the p53 protein incubated with JMJD6 (WT) (Figure 4A); no hydroxylation of p53 was detected when incubated with JMJD6 (H187R) (Figure 4B) via LC-MS/MS identification. To gain further insights into the influence of the mutation in JMJD6 to the p53 pathway, the expression of the mRNA and p21 protein was measured in HCT116 p53+/+ or HCT116 p53−/− cells. 

It is well-studied that the activated tumor suppressor p53 leads to the transient expression of downstream cyclin-dependent kinase inhibitor p21 [32]. Measurements of p21 by real-time RT-PCR and Western blotting experiments demonstrated that JMJD6 overexpression in HCT116 p53+/+ cells resulted in a reduced expression of mRNA (Figure 4C) and the p21 protein (Figure 4D) (*p* < 0.05), whereas, in HCT116 p53−/− cells, it showed no significant difference of p21 mRNA abundance between JMJD6 overexpression and JMJD6 (H187R) mutation (Figure 4C). In HCT116 p53+/+ and p53−/− cells, in contrast, the JMJD6 (H187R) mutation did not affect the expression of p21 (Figure 4C). These experiments depicted that the upregulation of p21 by JMJD6 was p53-dependent and indicated that JMJD6 (H187R) abolishes the lysyl-hydroxylation of p53; thus, p53 cannot inhibit the expression of p21.

## 3. Discussion

Our study provided a computational workflow integrating seven softwares to filter the deleterious nonsynonymous single nucleotide polymorphisms (nsSNPs) of JMJD6. Thirteen top disease-associated nsSNPs out of 255 nsSNPs retrieved from dbSNP for protein JMJD6 were predicted with high confidence, and the site H187R was identified to be more meaningful, taking the protein domain, sequence conservation, and three-dimensional structure, as well as damaging/deleterious predictions, into consideration. Biochemical experiments together with mass-spectrometry-based proteome validation demonstrated that the H187R mutation in JMJD6 reduced the modification of U2AF65 and p53, thus altering the mRNA splicing by U2AF65 and p53 transcriptional activities.

The effective filtration of deleterious nsSNPs benefits from several mature softwares and the remarkable principles of the appropriate integrated methods, as well as the biological filtrations. In recent years, a lot of software has been developed to predict deleterious nsSNPs through different algorithms and biological considerations, which is cost-effective and fast. At present, we integrated software for predicting damaging nsSNPs SIFT, PANTHER, and SNAP and software for inferring that the damaging effects of disease-related nsSNPs PhD-SNP, PolyPhen-2, SNPS&GO, and I-Mutant 2.0 are relatively mature, which has been widely employed in several previous studies [18,33,34,35,36]. The rationales of the prediction and inference of deleterious nsSNPs rely on considerations of the protein domain and sequence conservation. Furthermore, biological filtrations based on the protein domain, sequence conservation, and protein structure narrowed down the candidates to one site, which could not be achieved automatically through machine predictions.

JMJD6 is well-characterized as histone arginine demethylase and lysyloxidase to target histones and downstream substrates, including U2AF65 and p53. Although histones were known as JMJD6 substrates, a previous study based on subcellular localization and interaction analyses implied that histones are not a focus of Jmjd6 activity when interacting with Jmjd6 [8,37]. In our study, the regulation of p53 transcriptional activity and the alternative mRNA splicing pattern involved in the JMJD6 (H187R) mutation has been verified for the first time from the perspective of hydroxylation. For U2AF65, RT-PCR analyses detected the altered splicing pattern of the endogenous tumor antigen gene MIA2 after JMJD6 (H187R) abolished the lysyl-hydroxylation of U2AF65. For p53, the real-time RT-PCR and Western blotting experiments showed that JMJD6 (H187R) abolishes the lysyl-hydroxylation to p53, and thus, p53 cannot inhibit the p21 expression. In general, our experiments on the regulatory effect of JMJD6 (H187R) on its downstream genes complete our acknowledgment of JMJD6.

Meanwhile, several studies that attempted to predict the functional consequences of a nsSNP about whether it is disease-related or neutral were mainly based on computational software. Although nsSNPs are widely distributed in humans, with a total of over 3.1 million SNPs [38], only a couple of mutant proteins have been/could be detected at the expression level in human samples [25,39]. In our study, the MS-based identification and biochemical verification of this deleterious nsSNP remained necessary and meaningful, not only for confirming the accuracy of our workflow but also for providing an effective methodology for other researchers.

In conclusion, the combination of computational methods and biological viewpoints can accurately screen out the biological meaningful deleterious nsSNP (H187R) in JMJD6. Later biochemical experiments validated the site and proved that alterations of the site could influence the function of the downstream genes (U2AF65 and p53). Furthermore, our study provides an effective computational workflow for digging out the deleterious nsSNPs that influence downstream cellular processes for researchers.

## 4. Materials and Methods

### 4.1. Data Mining

All SNP information for JMJD6 was retrieved from the NCBI dbSNP (https://www.ncbi.nlm.nih.gov/snp/, accessed on 12 January 2019. The associated information about the SNPs was retrieved from UniProt (http://www.uniprot.org, accessed on 12 January 2019). The structural information of JMJD6 was obtained from the PDB (Protein Data Bank, http://www.rcsb.org/, accessed on 17 March 2019) with accession number 6FQC.

### 4.2. Prediction of Functional Consequences of Non-Synonymous Coding SNPs

There are numerous computational tools available to predict deleterious nsSNPs based on the changes in the structure and stability of the protein in a single-point mutation. We used SIFT, PANTHER, and PhD-SNP, which employ evolutionary sequence relationships, to identify the tolerance or intolerance, while PolyPhen-2, SNPS&GO, SNAP, and I-Mutant 2.0 used the structural and functional aspects of the protein.

### 4.3. Sequence Homology-Based Prediction of Damaging nsSNPs by SIFT

**SIFT** (**S**orting **I**ntolerant **F**rom **T**olerant) [40] predicts whether an amino acid substitution affects the protein function based on sequence homology and the physical properties of amino acids. SIFT can be applied to naturally occurring nonsynonymous polymorphisms and laboratory-induced missense mutations. Substitution scores above 0.05 are considered tolerant, whereas substitutions with scores below 0.05 are considered intolerant. SNPs with a SIFT score below 0.05 and marked with “AFFECT PROTEIN FUNCTION” are considered deleterious nsSNPs. The website of SIFT: http://sift.bii.a-star.edu.sg/ (accessed on 15 January 2019).

### 4.4. Structure Homology-Based Prediction of Damaging nsSNPs by PolyPhen-2

**PolyPhen-2** (**Poly**morphism **Phen**otyping v2) [41] is a tool that predicts the possible impact of an amino acid substitution on the structure and function of a human protein using straightforward physical and comparative considerations. It utilizes a machine-learning method combined with sequence alignment and protein structure information to predict deleterious nsSNPs. It then generates a **PSIC** score, and based on the difference of the PSIC score, it predicts the impact of amino acid substitution as benign, possibly damaging or probably damaging. Predicted SNPs marked with “probably damaging” are considered as deleterious nsSNPs. The website of PolyPhen-2: http://genetics.bwh.harvard.edu/pph2/ (accessed on 15 January 2019).

### 4.5. Functional Consequences Prediction Based on Neural Network Classification by SNAP

**SNAP** (**S**creening for **N**on-**A**cceptable **P**olymorphisms) [42] could potentially classify all nsSNPs in all proteins as non-neutral (effect on function) and neutral (no effect) using the sequence-based computationally acquired information alone. Predicted SNPs marked with “effect” are considered deleterious nsSNPs. The website of SNAP: https://www.rostlab.org/services/snap/ (accessed on 16 January 2019).

### 4.6. Protein Stability Changes Predicted by I-Mutant 2.0

**I-Mutant 2.0** [43] is a Support Vector Machine-based web server for the automatic prediction of protein stability changes upon single-site mutations. This tool can evaluate the stability change of single-site mutations starting from the protein structure or the protein sequence. When providing a protein three-dimensional structure, I-Mutant 2.0 can predict whether the protein mutation stabilizes or destabilizes with an accuracy above 80%. DDG (Gibbs free energy change) above 0 represents an increased protein stability, while DDG below 0 represents a decreased stability. Predicted SNPs marked with “Decrease Stability” are considered as deleterious nsSNPs. The website of I-Mutant 2.0: http://folding.biofold.org/i-mutant/i-mutant2.0.html (accessed on 27 January 2019).

### 4.7. Disease-Related Prediction of nsSNPs by PhD-SNP, SNPs&GO, and PANTHER

**PhD-SNP** (**P**redictor of **h**uman **D**eleterious **S**ingle **N**ucleotide **P**olymorphisms) [44] is based on a support vector machine (SVM)-based classifier. The reliability index value (RI) is used to evaluate the probability of the amino acid being deleterious. Predicted SNPs marked with “Disease” are considered deleterious nsSNPs. A probability > 0.5 is considered disease-associated, whereas ≤0.5 is considered neutral. The website of PhD-SNP: http://snps.biofold.org/phd-snp/phd-snp.html (accessed on 21 January 2019).

**SNPs&GO** [45] predicts the damaging SAPs (Single Amino acid Polymorphisms) using functional information codified by GO (Gene Ontology) terms. It classifies disease-associated SNPs by the SVM. The server has two components: one is sequence-based, and the other is structure-based. The RI value (reliability index) is evaluated as the potential if the predicted protein is stable. RI values higher than 0.5 are considered disease-associated SNPs. Predicted SNPs marked with “Decrease” are considered deleterious nsSNPs. The website of SNPs&GO: https://snps.biofold.org/snps-and-go/snps-and-go.html (accessed on 19 January 2019). 

**PANTHER** (**P**rotein **An**alysis **Th**rough **E**volutionary **R**elationships) [46] estimates the likelihood of a particular nonsynonymous coding SNP to cause a functional impact on the protein. A substitution position-specific evolutionary conservation (subPSEC) score was calculated for the estimation probability by the Hidden Markov Model. The higher the subPSEC score, the more likely it is to be a deleterious nsSNP. Predicted SNPs marked with “probably damaging” are considered deleterious nsSNPs. The website of PANTHER for coding SNP analyses: http://www.pantherdb.org/tools/csnpScoreForm.jsp (accessed on 19 January 2019).

### 4.8. Plasmids, Antibodies, and Reagents

The cDNA for wild-type JMJD6 was amplified by PCR and ligated into Xba I/EcoR I sites of the pcDNA3.1 vector that contains one or three copies of FLAG. pcDNA3.1-U2AF65 and pcDNA3.1-p53 were kept in our lab. The GST-JMJD6, GST-U2AF65, and GST-p53 expression plasmids were constructed by cloning full-length cDNA into the pGEX-4T-3 vector. The JMJD6 (H187R) mutant was generated by site-directed mutagenesis. All the clones were confirmed by DNA sequencing. The sources of antibodies against the following proteins were as follows: β-actin (SAB3500350) from Sigma (Sigma-Aldrich, Inc., St. Louis, MO, USA), JMJD6 (sc-28348) from Santa Cruz (Santa Cruz Biotechnology, Inc., Santa Cruz, CA, USA), p53 monoclonal antibody (K0181-3), and p21 (K0081-3) from MBL (Medical & Biological Laboratories Co., Ltd, Tokyo, Japan). The source of the chemical reagent was as follows: DTT (dithiothreitol, 1758-9030) was purchased from INALCO (Inalco Pharmaceuticals, San Luis Obispo, CA, USA) and IAA (Iodoacetamide, SLCC6164) from Sigma (Sigma-Aldrich, Inc., St. Louis, MO, USA).

### 4.9. Cell Culture and Transfection

The HeLa, HCT116 p53+/+, and HCT116 p53−/− cells were kept in our lab and maintained in Dulbecco’s Modified Eagle’s Medium (DMEM) (Hyclone) supplemented with 10% fetal bovine serum (FBS). All transfections were carried out using Lipofectamine 3000 (Invitrogen).

Briefly, the cells were first incubated in serum-free medium for 1 h. Next, the Lipo3000 reagent was diluted in serum-free medium and incubated for 5 min. Simultaneously, P3000 and 3μg of plasmid were also diluted with serum-free medium. Then, the plasmid solution was added to the Lipo3000 dilution and incubated for 25 min. The mixed solution containing the plasmid and Lipo3000 was added to the cells. After incubation for 6 h, the medium was changed. Samples were collected after 48 h of incubation.

### 4.10. Hydroxylation Assay

GST-U2AF65 was incubated with GST-JMJD6 or GST-JMJD6 (H187R) in the presence of 2-OG (500 µM) and Fe(II) (400 µM) for 2 h at 37 °C. 

### 4.11. SDS-PAGE and In-Gel Digestion

The hydroxylation assay mixture was separated by 10% SDS-PAGE and stained with CBB. SDS-PAGE revealed 2 protein bands with the weight about 55 kDa (TP53, 53 kDa; U2AF65, 54 kDa) from 4 samples. These bands were then cut out (from 55 kDa to 70 kDa). The gel was washed with washing buffer (8-mg/mL ammonium bicarbonate and 50% ACN) several times until the color was gone, avoiding the gel dry through the washing progress. The gel was dehydrated with ACN and incubated with DTT (Dithiothreitol) solution (10-mM DTT in 20-NH_4_HCO_3_) for half an hour at 57 ℃. The DTT solution was removed from the gel. The gel was dehydrated and covered with 55-mM IAA (Iodoacetamide) solution and incubated at 37 °C for 15 min. The IAA solution was removed, and the gel was dehydrated again and covered with trypsin solution (0.01 mg/mL in 25-mM NH_4_HCO_3_) overnight. The peptides were extracted from each gel band with 50 μL of 50% ACN containing 0.1% FA.

The peptides extracted from the gel were analyzed using Thermo Scientific Q Exactive HF.

### 4.12. Western Blot

The protein was separated by 10% SDS-PAGE at 100 V in running buffer and then transferred onto a nitrocellulose membrane at 300 mA for 1.5 h. After the protein was blocked by 5% BSA for one hour, the primary antibody was incubated overnight with a dilution ratio of 1:1000 at 4 °C. Subsequently, the membrane was washed 3 times with TBST (Tris-Buffered Saline with Tween) and incubated with the secondary antibody at a dilution ratio of 1:5000 for one hour. Finally, the protein was detected by chemiluminescence and autoradiography.

### 4.13. Real-Time Reverse Transcription PCR

Total cellular RNAs were isolated with the TRIzol reagent (Invitrogen) and used for first strand cDNA synthesis with the Reverse Transcription System (Promega, A3500). Quantitation of all the gene transcripts was done by qPCR using a Power SYBR Green PCR Master Mix and an ABI PRISM 7300 sequence detection system (Applied Biosystems, Foster City, CA, USA), with the expression of GAPDH as the internal control. The primer pairs used were as follows: p21 forward primer, 5′-CATCCCGTGTTCTCCTTT-3′; p21 reverse primer, 5′-GTGCCATCTGTT TACTTCTCA-3′; p53 forward primer, 5′-GTTCCGAGAGCTGAATGAGG-3′; p53 reverse primer, 5′-TCTGAGTCAGGCCCTTCTGT-3′; GAPDH forward primer, 5′-CCCACTCCTCC ACCTTTGAC-3′; GAPDH reverse primer, 5′-CATACCAGGAAATGAGCTTGACAA-3′; MIA2-E18 primer, 5′-CTGAAACAGAGCTTAAATTTGAAC-3′; and MIA2-E20 primer, 5′-CTGGCGGAGGAAACATCATCC-3′.

### 4.14. Protein Identification and Quantification

All the raw data were processed by Peaks online (Bioinformatics Solutions Inc., Waterloo, ON, Canada). All the data were searched against the Swiss-Prot human database (20,379 entries). The search parameters were set as the following: the precursor mass tolerance was set at 15 ppm, and the fragment mass tolerance was set at 0.05 Da; cysteine carbamidomethylation was set as a fixed modification, and N-terminal acetylation, lysine hydroxylation, arginine methylation, and deamidation for the N-terminal and glutamine were set as variable modifications. The false discovery rate was set to 0.01 for both proteins and PSM with a minimum length of six amino acids. A maximum of three missed cleavages was allowed for the database search.

## Figures and Tables

**Table 1:** Twenty-eight nsSNPs were predicted by SIFT, PolyPhen-2, SNAP, I-Mutant, PhD-SNP, PANTHER, and SNP&GO.

SNP ID	Amino Acid Change	SIFT	PolyPhen	SNAP	I-MUTANT	PhD-SNP	PANTHER	SNPs&GO
rs770686748	R411W	D	PD	D	D	N	—	N
rs759427088	G409E	D	PD	D	D	N	N	N
rs769402176	R399H	D	PD	D	D	N	N	N
rs1177861863	D393H	D	PD	D	D	N	N	N
rs751792177	R373H	D	PD	D	D	D	D	N
rs757164575	S352C	D	PD	D	D	N	D	N
rs1157910263	S340C	D	PD	D	D	N	D	N
rs1417542107	I284T	D	PD	D	D	D	D	D
rs778790592	P268T	D	PD	D	D	D	D	D
rs778790592	P268A	D	PD	D	D	D	D	D
rs1418743067	F266S	D	PD	D	D	D	D	D
rs374399276	R242W	D	PD	D	D	D	D	D
rs765486191	R205H	D	PD	D	D	D	N	N
rs766654214	R205C	D	PD	D	D	D	D	N
rs758184469	K204E	D	PD	D	D	D	D	D
rs1159480887	H187R	D	PD	D	D	D	D	D
rs1366225731	R181C	D	PD	D	D	D	D	D
rs1162409498	D159N	D	PD	D	D	D	D	D
rs1381511354	F156S	D	PD	D	D	D	D	D
rs748652403	D149V	D	PD	D	D	N	N	N
rs369981508	P129L	D	PD	D	D	D	D	D
rs1301580761	D126G	D	PD	D	D	D	N	N
rs1394232718	Y117H	D	PD	D	D	D	D	N
rs1398491957	F99C	D	PD	D	D	D	D	D
rs746020005	Y94C	D	PD	D	D	D	N	N
rs750848447	R28W	D	PD	D	D	D	—	N
rs1490052400	S23W	D	PD	D	D	D	—	N
rs1278674934	R8C	D	PD	D	D	N	—	N

N: Neutral, D: Deleterious, and PD: probably damaging. —: not aligned. nsSNPs labeled in red are filtered by a combination of all the computational predictions.

## Data Availability

Data are available upon requests.

## Supplementary Materials

The following are available online. Figure S1: Sequence conservation across species. Figure S2: Protein structure homology modeling of JMJD6. Figure S3: SDS-PAGE gel image. Table S1: List of nsSNPs in JMJD6 and the results of the computational analysis. Table S2: List of the identified peptides.

## Sample Availability

Samples are available from the authors.
