# Supplementary material for: Computational and Mass Spectrometry-Based Approach Identify Deleterious Non-Synonymous Single Nucleotide Polymorphisms (nsSNPs) in JMJD6
Source: Molecules. 2021 Jul 31;26(15):4653. doi: 10.3390/molecules26154653 (PMC8347302; doi:10.3390/molecules26154653)
Supplement: Supplementary file 1 [file molecules-26-04653-s001.zip › molecules-1232771-supplementary.pdf]

Supplementary Information

Computational and mass spectrometry-based approach identify deleterious non-synonymous single nucleotide polymorphisms (nsSNPs) in JMJD6

Tianqi Gong, Lujie Yang, Fenglin Shen, Hao Chen, Ziyue Pan, Quanqing Zhang, Yan Jiang, Fan Zhong, Pengyuan Yang and Yang Zhang

1. Department of Systems Biology for Medicine, and Institutes of Biomedical Sciences, Shanghai Medical College, Fudan University, Shanghai, China.
2. College of Bioscience and Biotechnology, Yangzhou University, Yangzhou, Jiangsu, 225009, China.
3. Shanghai Stomatological Hospital, Fudan University, Shanghai, China.
4. Department of Chemistry, University of California, Riverside, California 92521, United States.

Table of contents

1. Supplementary materials and methods

LC-MS/MS Analysis for Proteomic Analysis

For proteome experiments, LC-MS/MS analysis was performed using an Easy-nLC 1000 nanoflow LC system (Thermo Fisher Scientific) connected to a Q Exactive HF mass spectrometer (Thermo Fisher Scientific, Rockford, IL, USA). The peptide mixture was then resuspended in Solvent A (0.1% formic acid in water) for LC-MS/MS analysis. The obtained peptides were separated on a 150 μm × 12 cm silica microcolumn (particle size, 1.9 μm; pore size, 120 Å; SunChrom, USA) at a flow rate of 600 nl/min. The gradient was set 5–35% mobile phase B (0.1% formic acid in acetonitrile) for 75 min. The MS analysis for QE HF was performed with one full scan (300–1400 m/z, R = 60,000 at 200 m/z) at automatic gain control target of 3e6 ions, followed by up to 20 data-dependent MS/MS scans. The AGC was set as 5e4 ions with a max injection time of 40 ms. A 1.6 m/z isolation window was used. The normalized collision energy was 27% and the resolution was 15,000 at 200 m/z.

2. Supplementary figure titles and legends

Figure S1. Multiple sequence alignment of JMJD6 protein.

Consensus scores are shown in the bottom row. Amino acids in white and labeled with red background implies completely aligned across all species. Amino acids labeled in red represent highly conserved across species.

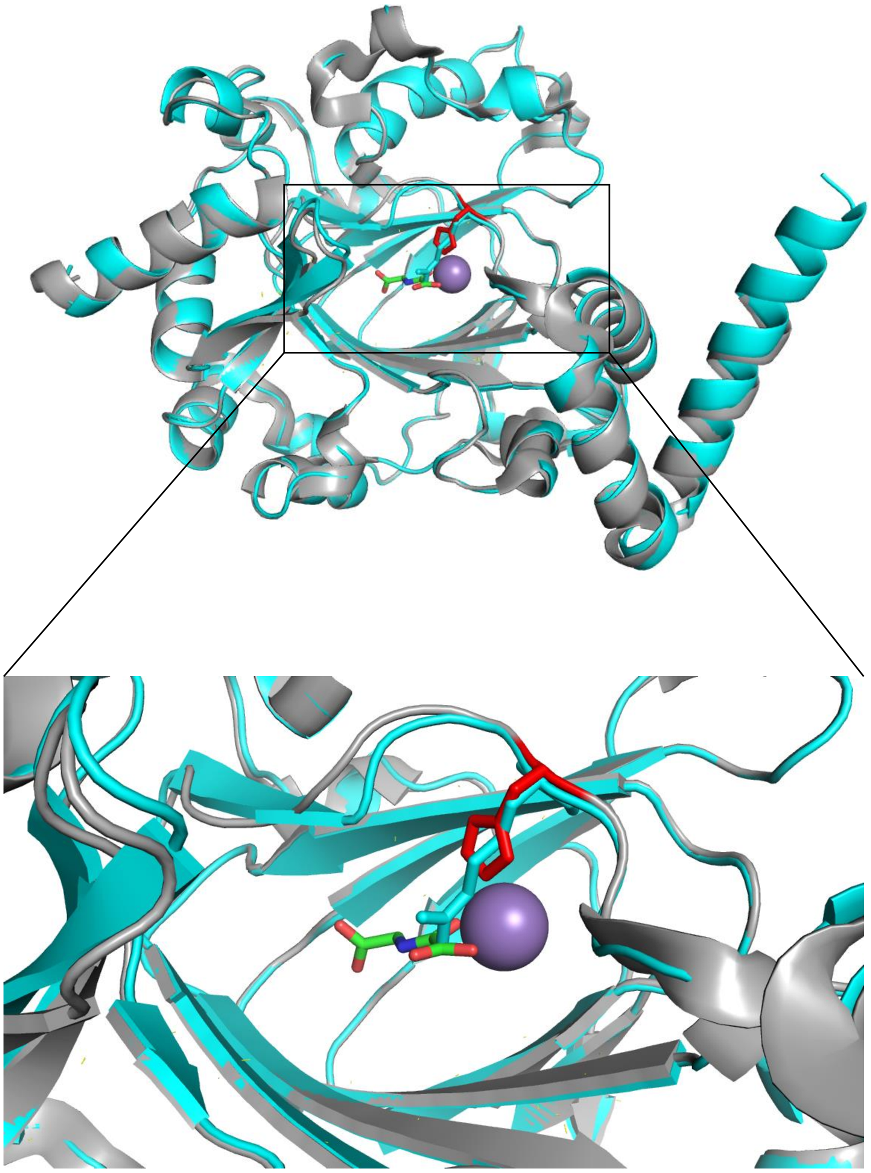

Figure S2 Protein structure homology-modeling of JMJD6.
Protein structure homology-modeling of the JMJD6 (H187R) mutation and the WT. Grey cartoon plot represents WT, blue cartoon plot represents JMJD6 (H187R) mutation. Purple ball represents Fe(II).

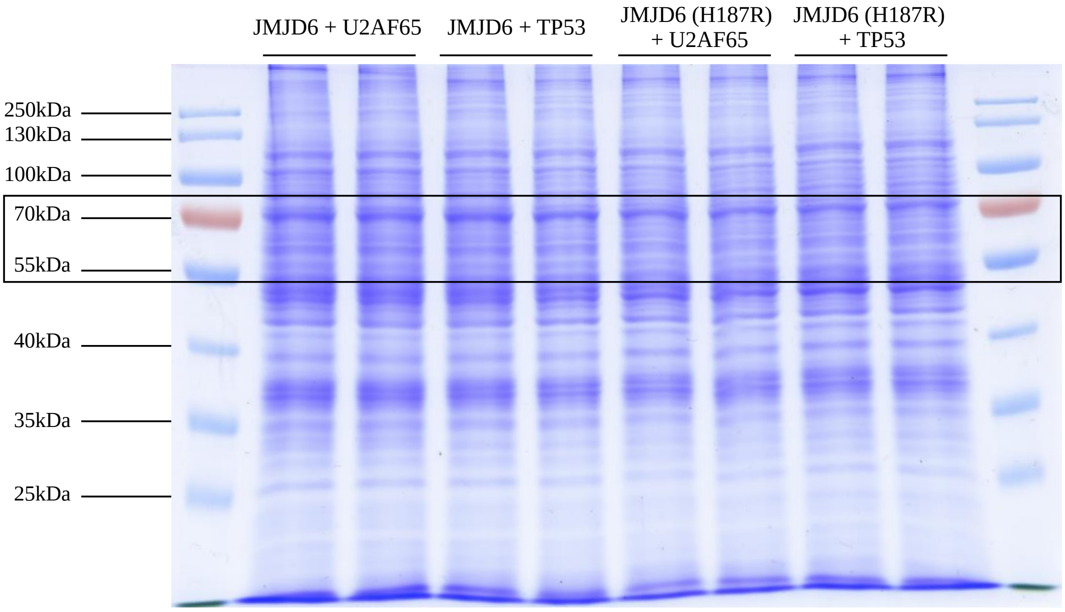

Figure S3 SDS-PAGE gel image.

Gel image showing the location of gel slices sample1-4 by in-gel digestion and the corresponding counterpart 1-4 region processed by the whole-gel procedure for Hela cell lysate with over-expressed JMJD6 (additional of U2AF65 and TP53), or with overexpressed JMJD6 (H187R) (additional of U2AF65 and TP53).

3. Supplementary table titles

Table S1. List of nsSNPs in JMJD6 gene and the results of computational analysis.

Table S2. List of identified peptides
